# Targeted Therapy for Older Patients with Non-Small Cell Lung Cancer: Systematic Review and Guidelines from the French Society of Geriatric Oncology (SoFOG) and the French-Language Society of Pulmonology (SPLF)/French-Language Oncology Group (GOLF)

**DOI:** 10.3390/cancers14030769

**Published:** 2022-02-02

**Authors:** Laurent Greillier, Manon Gauvrit, Elena Paillaud, Nicolas Girard, Coline Montégut, Rabia Boulahssass, Marie Wislez, Frédéric Pamoukdjian, Romain Corre, Mathilde Cabart, Philippe Caillet, Yaniss Belaroussi, Matthieu Frasca, Pernelle Noize, Pascal Wang, Soraya Mebarki, Simone Mathoulin-Pelissier, Anne-Laure Couderc

**Affiliations:** 1 Multidisciplinary Oncology and Therapeutic Innovations Department, AP-HM Marseille France, Aix-Marseille University, CNRS INSERM CRCM, F-13009 Marseille, France; laurent.greillier@ap-hm.fr; 2 French-Language Society of Pulmonology (SPLF), F-75006 Paris, France; nicolas.girard2@curie.fr (N.G.); marie.wislez@aphp.fr (M.W.); 3 French-Language Oncology Group (GOLF), F-75006 Paris, France; 4 Inserm CIC1401 Institut Bergonié Clinical Research Plateform for Older Patients with Cancer (PACAN), F-33076 Bordeaux, France; m.gauvrit@bordeaux.unicancer.fr (M.G.); yaniss.belaroussi@u-bordeaux.fr (Y.B.); matthieu.frasca@chu-bordeaux.fr (M.F.); pernelle.noize@chu-bordeaux.fr (P.N.); simone.pelissier@chu-bordeaux.fr (S.M.-P.); 5 Department of Geriatrics Geriatric Oncology Clinic HEGP Paris Cancer Institute CARPEM, AP-HP, F-75015 Paris, France; elena.paillaud@aphp.fr (E.P.); philippe.caillet@aphp.fr (P.C.); soraya.mebarki@aphp.fr (S.M.); 6 Faculty of Health, Université Paris, F-75006 Paris, France; 7 French Society of Geriatric Oncology (SoFOG), F-63122 Ceyrat, France; coline.montegut@ap-hm.fr (C.M.); boulahssass.r@chu-nice.fr (R.B.); frederic.pamoukdjian@aphp.fr (F.P.); romain.corre@ch-cornouaille.fr (R.C.); 8 Thorax Institute Curie-Montsouris, F-75014 Paris, France; 9 UVSQ Paris, F-78000 Saclay, France; 10 Internal Medicine Geriatrics and Therapeutic Unit AP-HM, F-13009 Marseille, France; 11 Coordination Unit of Geriatric Oncology Rehabilitation and Autonomy Department University Hospital of Nice, F-06000 Nice, France; 12 Nice Sophia Antipolis, University Onco-Age, F-06100 Nice, France; 13 Oncology Thoracic Unit Pulmonology Department AP-HP Hôpital Cochin, F-75014 Paris, France; pascal.wang@aphp.fr; 14 Paris University Centre de Recherche des Cordeliers, Sorbonne University INSERM Team Inflammation Complement and Cancer, F-75006 Paris, France; 15 Geriatric Department APHP, Avicenne Hospital, F-93000 Bobigny, France; 16 I SERM UMR_S942 Cardiovascular Markers in Stressed Conditions MASCOT, Sorbonne Paris Nord University, F-93000 Bobigny, France; 17 Pulmonology Department Cornouaille Hospital, F-29000 Quimper, France; 18 Oncology Department Institut Bergonié, F-33076 Bordeaux, France; m.cabart@bordeaux.unicancer.fr; 19 INSERM U955 IRMB Université Paris-Est, F-94000 Créteil, France; 20 Department of Thoracic Surgery Haut-Leveque Hospital Bordeaux University, University of Bordeaux, F-33000 Bordeaux, France; 21 INSERM Bordeaux Population Health Research Center EPICeNE Team UMR 1219, F-33000 Bordeaux, France; 22 Palliative Medicine Department CHU, F-33000 Bordeaux, France; 23 Bordeaux Population Health Centre Recherche U1219, Equipe Cancer et Environnement EPICeNE, Bordeaux University, F-33000 Bordeaux, France; 24 Clinical Pharmacology Department CHU Bordeaux, F-33000 Bordeaux, France; 25 Univ Bordeaux INSERM BPH U1219 Team Pharmaco-Epidemiology, F-33000 Bordeaux, France; 26 CNRS EFS ADES Aix-Marseille University, F-13015 Marseille, France

**Keywords:** lung cancer, targeted therapy, efficacy, toxicity, safety, quality of life, geriatric assessment

## Abstract

**Simple Summary:**

Targeted therapy has become essential in the treatment of non-small cell lung cancer (NSCLC). There are currently no guidelines for older patients who are frailer with regard to this type of treatment. Two learned societies, the French Society of Geriatric Oncology (SoFOG) and the French-language Society of Pulmonology (SPLF)/French-language Oncology Group (GOLF), joined forces to conduct a systematic review of the literature from May 2010 to May 2021 regarding the efficacy, toxicity, and feasibility of targeted therapy in older patients with NSCLC. Guidelines were then drawn up to enable clinicians to adapt the type of targeted therapy proposed according to the oncological and geriatric profile of the older patient with NSCLC.

**Abstract:**

Systematic molecular profiling and targeted therapy (TKI) have changed the face of Non-Small Cell Lung Cancer (NSCLC) treatment. However, there are no specific recommendations to address the prescription of TKI for older patients. A multidisciplinary task force from the French Society of Geriatric Oncology (SoFOG) and the French Society of Pulmonology/Oncology Group (SPLF/GOLF) conducted a systematic review from May 2010 to May 2021. Protocol registered in Prospero under number CRD42021224103. Three key questions were selected for older patients with NSCLC: (1) to whom TKI can be proposed, (2) for whom monotherapy should be favored, and (3) to whom a combination of TKI can be proposed. Among the 534 references isolated, 52 were included for the guidelines. The expert panel analysis concluded: (1) osimertinib 80 mg/day is recommended as a first-line treatment for older patients with the EGFR mutation; (2) full-dose first generation TKI, such as erlotinib or gefitinib, is feasible; (3) ALK and ROS1 rearrangement studies including older patients were too scarce to conclude on any definitive recommendations; and (4) given the actual data, TKI should be prescribed as monotherapy. Malnutrition, functional decline, and the number of comorbidities should be assessed primarily before TKI initiation.

## 1. Introduction

Lung cancer is a disease of older adults with a median age of around 70 years at the time of diagnosis [1,2] and is the leading cause of cancer death, particularly in the USA and Europe and in the elderly population [3]. However, there are very few specific guidelines relating to the treatment of lung cancer in older patients due to their under-representation in clinical trials [4]. As a result, older patients are treated with therapies of which the safety in use, efficacy, toxicity, and dose are based on clinical trials conducted on patients that do not reflect the older population. With age, pharmacokinetic changes [5] and frailty/comorbidities, which sometimes go hand in hand with polypharmacy, can modify the tolerance and efficacy of the molecules in this older population [6,7,8,9,10]. However, treatment compliance is essential to ensure treatment efficacy and avoid jeopardizing the outcome [10]. In the past ten years, the therapeutic strategy for the treatment of primary metastatic bronchial cancer has progressed rapidly, particularly with the emergence of targeted therapy as tyrosine kinase inhibitors (TKI). Systematic molecular profiling has revolutionized the treatment of patients with a genomic alteration by proposing therapy targeting the most common oncogene addiction pathways, such as EGFR, ALK, or ROS1. In recent years, numerous other oncogenic drivers that can be targeted have emerged such as BRAF, MET, RET, HER2, KRAS, NTRK, or NRG1. However, few studies have investigated these oncogenic addictions in the treatment of NSCLC [11]. According to the literature, molecular panels are proposed less frequently in older patients compared to younger patients [12]. EGFR activating mutations are found in approximately 10% of Caucasian patients [13]. A study on patients aged 80 years has shown that EGFR mutations are found in similar proportions to those in younger patients [14]. The following drugs are used for TKI in the treatment of patients with metastatic non-small cell lung cancer (NSCLC) with an EGFR mutation: gefitinib and erlotinib, which are first generation TKI, afatinib (2nd generation), dacomitinib (2nd generation), and osimertinib (3rd generation). ALK rearrangements are detected in 3% to 5% of patients with NSCLC [15,16,17,18]. Crizotinib (1st generation), ceritinib, alectinib, brigatinib (all 2nd generation inhibitors), and lorlatinib (3rd generation) are used in the treatment of advanced cancer with an ALK rearrangement. ROS1 rearrangements are rare (approximately 1% of NSCLC) and are primarily diagnosed at a young age [14]. Today, crizotinib has a marketing authorization as a first-line treatment in patients with metastatic NSCLC with a ROS1 rearrangement. A combination of dabrafenib and trametinib is indicated as a first-line treatment in patients with advanced NSCLC with a BRAF V600E mutation. Mutations of the splice site of MET exon 14 are quite rare, found in 3% to 4% of NSCLC [19]. MET mutations seem to be more common in older subjects [20,21,22].

The aim of the joint recommendations from the two learned societies that are the French Society of Geriatric Oncology (SoFOG) and the French-language Society of Pulmonology (SPLF)/French-language Oncology Group (GOLF) is to compile, from a literature review, data regarding the indication, efficacy, and tolerance of the TKI used in patients over 65 years with stage II, III, and IV NSCLC. The primary objective is to determine which older patients with NSCLC can be offered TKI. The secondary objectives are to decide for which older patients with NSCLC should monotherapy (TKI alone) or a combination of TKI be considered.

## 2. Materials and Methods

This systematic literature review has been registered in PROSPERO (www.crd.york.ac.uk/PROSPERO/display_record.asp?ID=CRD42021224103, accessed on 25 January 2022). Each strategy followed a standardized, declared protocol.

### 2.1. Research Questions

The research questions were formulated by a multidisciplinary group (pulmonary oncologist, geriatrician and methodologist) and are listed below:

(1) For which older patients with non-small cell lung cancer (NSCLC) can we propose the following TKI: EGFR tyrosine kinase inhibitors, ALK tyrosine kinase inhibitors, ROS1 tyrosine kinase inhibitors, and inhibitors of other molecular alterations BRAF/MET?
(2) For which older patients with NSCLC must we consider mono-therapy (TKI alone)?
(3) For which older patients with NSCLC can we consider a combination of several TKI?

### 2.2. Inclusion and Exclusion Criteria

The articles included were (1) trials or observational studies or systematic reviews or meta-analyses, (2) stage II, III, or IV NSCLC, (3) older patients (65 years or over, 70 years or over, 75 years or over, or 80 years or over) mentioned in the title or abstract, (4) outcomes: Overall survival (OS), progression-free survival (PFS), disease-free survival (DFS), treatment and/or treatment feasibility and/or treatment complications and/or toxicity, quality of life, G8 screening score, or data from Comprehensive Geriatric Assessments (CGA).

We chose to exclude narrative reviews, case series, clinical cases, comments, and opinions.

### 2.3. Data and Research Algorithm

The research was conducted between May 2010 and May 2020. The literature was monitored until the paper was written (May 2021). Medline, Cochrane Database, and Embase were queried. The MeSH terms used are listed in the Supplementary Data (Figure S1).

### 2.4. Article Selection

A group comprising a pulmonary oncologist, a geriatrician, and a methodologist independently read the titles and abstracts of the articles included and coded the references “yes” (to be included), “no” (not to be included), or “uncertain”. Any discrepancies, particularly regarding “uncertain” articles, were discussed at a consensus meeting. All decisions made by the members of the coordination group, and, more particularly, the discrepancies and references included, were documented. The selected articles were divided equally between five work groups, each comprising a (pulmonary) oncologist, a geriatrician, and a methodologist. They read all the articles assigned and decided whether they should be included in the review or not, the data to be studied, and any study bias. All the decisions made by the members of the work group were documented. A standardized grid was created. To assess bias, the Cochrane grid was used for randomized trials [23]. The study selection process presented above is documented in a flow diagram based on the PRISMA (Preferred Reported Items for Systematic Reviews and Meta-Analyses) reporting guidelines endorsed by INESSS [24]. Ranking of guidelines are based on HAS 2013, and a descriptive table of the recommendation staging (grade A, B, or C) is available in Table S1) [25].

## 3. Results

**Figure 1:**
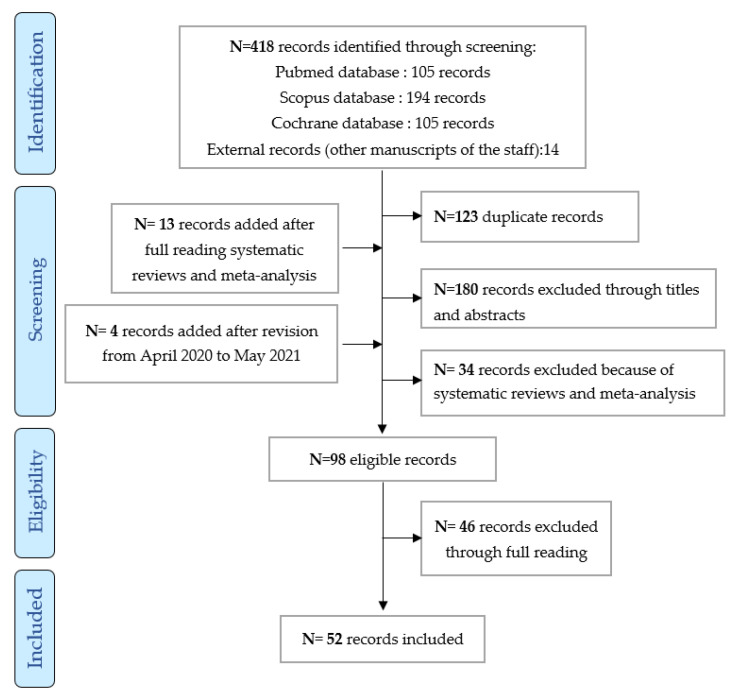
Inclusion diagram of the studies analyzed from May 2010 to May 2021.

The literature search identified 534 references in three databases (Pubmed, Cochrane, and Scopus) and 14 external references (articles previously published by members of this work group). A further 295 abstracts and 98 articles to be read by the groups were identified after full reading meta-analysis and reviews and after revision between April 2020 and May 2021. Ultimately, 52 articles were included (cf. flow chart in Figure 1).

To answer the 3 questions predefined in the methodology, we included:

- 28 articles on the efficacy of EGFR TKI (only articles mentioning the mutation were included: 17 prospective observational and retrospective non-randomized studies dedicated to older subjects [26,27,28,29,30,31,32,33,34,35,36,37,38,39,40,41,42], and 11 prospective randomized or non-randomized studies not dedicated to older subjects [43,44,45,46,47,48,49,50,51,52,53]; 5 articles on the efficacy of ALK TKI were analyzed: 2 prospective non-randomized observational studies not dedicated to older subjects (subgroup of older subjects) and 3 randomized prospective studies not dedicated to older subjects (subgroup of older subjects) [16,17,54,55,56]; only one study on the efficacy of ROS1 TKI as crizotinib was included [57].
- 36 articles on toxicity: 26 retrospective or prospective studies specific to older subjects [26,27,28,29,30,31,32,33,34,35,36,37,38,39,40,42,58,59,60,61,62,63,64,65,66,67], 5 randomized studies dedicated to older subjects [7,68,69,70,71], 4 prospective studies with a subgroup of older subjects [55,72,73,74], and a randomized study including a subgroup of older subjects [44].
- 36 articles on feasibility: 26 prospective and retrospective studies, 4 prospective studies with a subgroup of older subjects, 5 randomized studies specific to older subjects, and a randomized study with a subgroup of older subjects.
- four articles on quality of life [31,33,69,75] and 7 studies on geriatric data [36,41,42,66,71,74,76].

### 3.1. Efficacy

In the literature, the number of studies in older patients with dabrafenib/trametinib/vemurafenib/capmatinib/merestinib/savolitinib/lorlatinib/nintedanib/tepotinib/repotrectinib/pralsetinib/selpercatinib/alectinib/brigatinib/entrectinib/larotrectinib were limited.

#### 3.1.1. EGFR Tyrosine Kinase Inhibitors

Prospective and retrospective cohort studies were specifically dedicated to older subjects (≥65 years) (Table 1).

In older patients treated with osimertinib (80 mg/day [38,39,40]), the median overall survival varied from 19.4 to 38.6 months. In another objective response rate (ORR) varied from 56.5% to 61%, the median progression-free survival (PFS) varied from 6.4 to 17.7 months, and, in Furuta et al. [37], no significant difference was observed for PFS and OS between older subjects and younger subjects treated with osimertinib. In older patients treated with gefitinib (250 mg/day) [29,30,31,34,36], the ORR varied from 45.5% to 74.2%, PFS varied from 10.0 to 14.3 months, and OS varied from 19 to 33.8 months. PFS with gefitinib was homogenous (varying from 10.0 to 14.3 months), but OS varied due to the various regimens ranging from first-line therapy to other advanced lines of treatment. The same applied to osimertinib, with which PFS varied due to the different regimens; most of the studies included were anterior to the use of osimertinib as a first-line treatment.

For erlotinib (150 mg/day) [41,42], the ORR varied from 56.3% to 60.0%, and PFS varied from 9.3 to 15.5 months. 

For afatinib (40 mg/day) [28], the median PFS and OS were longer in patients with a lower dose (30 patients) compared to the other patients (8 patients) (PFS: 16.9 versus 3.5 months, HR 0.2, *p* = 0.001; OS: NR (not reached) versus 10.3 months, HR 0.09, *p* < 0.001). For a dose of 30 mg/day of afatinib [26,27], the ORR was 72.5% and PFS 12.9 months. The results are hard to interpret as there are very few studies assessing TKI as a first-line treatment. Osimertinib was not superior to the other TKI in the different studies. OS with osimertinib seemed to be shorter in these studies than the general population, but patients were mainly on second-line therapy or beyond. There is very little data for older patients with a Performance Status (PS) ≥ 2.

Prospective randomized or non-randomized studies not dedicated to older subjects (subgroup of older subjects) are shown in Table 2.

In older patients treated with osimertinib (80 mg/day) [51], a lower risk of progression was observed (similar to younger patients; HR 0.38). Two phase 3 studies [49,50] comparing osimertinib (80 mg/day) and gefitinib (250 mg/day) or erlotinib (150 mg/day) reported similar benefits in terms of PFS and OS with osimertinib in older subjects (as with younger subjects). In older patients treated with gefitinib (250 mg/day) or afatinib (40 mg/day), in three studies [43,44,53], PFS was in favor of afatinib in the group of subjects ≥ 65 years [43], and PFS and OS were better with afatinib compared to gefitinib for patients ≥ 75 years (as with younger patients) [44]. In Sequist et al. [45] (afatinib 40 mg/day versus chemotherapy), PFS (HR 0.64, 95% CI, 0.39 to 1.03, *p* = 0.58) favored afitinib for patients < 65 years, as well as patients ≥ 65 years. In two studies [47,48], PFS favored erlotinib (150 mg/day versus chemotherapy) as with younger patients. Comparing dacomitinib (45 mg/day) with gefitinib (250 mg/day), PFS favored dacomitinib for patients ≥ 65 years (HR 0.69, 95% CI, 0.48 to 0.99), as with patients < 65 years [46].

Thus, in patients with an EGFR activating mutation, the efficacy of anti-EGFR TKI in terms of ORR, PFS, and OS seems similar in patients aged ≥ 65 years (even over 75 years) and younger patients. In older patients, all lines and molecules included, ORR varied from 45.5% to 75.7%, PFS varied from 6.4 to 22 months, and OS varied from 19 to 38.6 months. PFS and OS were in favor of osimertinib as a first-line treatment in older subjects (as with younger subjects) compared to first generation TKI (erlotinib and gefitinib). In the case of a T790M mutation, osimertinib was more effective as a second-line treatment than platinum-based chemotherapy in terms of PFS (no data on OS). However, this greater efficacy needs to be weighed up against the toxicity results of osimertinib in this older population. There are probably few indications for the use of first generation TKI in the case of an EGFR mutation: Erlotinib and afatinib are more effective as first-line treatments than platinum-based chemotherapy in terms of PFS whatever the age (no data on OS). Afatinib is more effective than gefitinib as a first-line treatment in terms of PFS and OS, with an efficacy that seems greater in patients who had a decrease in dose. Dacomitinib is more effective than gefitinib as a first-line treatment in terms of PFS only but is not very pertinent in clinical practice.

#### 3.1.2. ALK Tyrosine Kinase Inhibitors in Older Subjects 

In two randomized phase 3 studies with a subgroup of patients aged ≥65 years and ≥75 years [16,54], a better PFS was obtained in the alectinib arm (300 mg twice/day or 600 mg twice/day) compared to crizotinib (250 mg twice/day). A Phase 1 study [56] including treatment with crizotinib (250 mg twice/day) showed an ORR very similar to that of younger patients. Another observational study comparing crizotinib, ceritinib, and alectinib [55] revealed similar PFS and OS with no significant difference in patients ≥65 years versus patients <65 years. Finally, an article comparing ceritinib with chemotherapy (cisplatine and pemetrexed) [17] showed PFS in favor of ceritinib (750 mg/day), as with younger patients. However, the results must be weighed up against the fact that, from an epidemiological viewpoint, very few older patients have the ALK mutation (3% to 5% of patients with NSCLC) (Table 3).

It is difficult to draw any conclusions due to the low number of studies dedicated to older subjects. Nevertheless, the efficacy of anti-ALK TKI in terms of ORR, PFS, and OS seems similar in patients aged 65 years and over and in younger subjects. In terms of PFS, alectinib is more effective than crizotinib as a first- or second-line treatment, whatever the age. In terms of PFS, ceritinib is more effective than platinum-based chemotherapy drugs as a first-line treatment, whatever the age (no data on OS).

#### 3.1.3. ROS1 Tyrosine Kinase Inhibitors in Older Subjects, Case of the Crizotinib

We found one non-randomized observational phase 2 study [57] including 127 patients of Asian origin with a ROS1 mutation treated with crizotinib (250 mg twice/day) as a fourth-line treatment or no longer receiving treatment. The study was conducted from 2013 to 2015, and the median age was 51.5 years. Twenty-one patients (16.5%) were ≥65 years, and the ORR was 61.9% (95% CI, 38.4 to 81.9), which is 13 out of 21 patients. In comparison, the ORR for younger patients was 73.6%. Age had no significant effect on clinical efficacy, and there is no argument to consider that the efficacy of crizotinib in patients with an ROS1 rearrangement would differ depending on age.

#### 3.1.4. Erlotinib Combined with Bevacizumab in Older Subjects

A randomized, multicentric phase 2 study [66] was carried out in Japan from 2015 to 2018 on 25 patients ≥75 years treated with a combination of erlotinib (150 mg/day) and bevacizumab as a first-line treatment. There was a partial response in 88% of cases (95% CI, 74 to 99), stability in 12% of cases, and, therefore, control of the disease in 100% of cases (95% CI; 88.7 to 100%). PFS was 12.6 months (95% CI; 8.0 to −33.7), and the median survival was not reached (OS NR; 95% CI; 34.0 months-NR). There is not enough data to draw up specific recommendations (only one phase 2 study). The results need to be weighed up against the toxicity induced by bevacizumab.

### 3.2. Toxicity 

Table 4 and Table 5.

#### 3.2.1. EGFR Tyrosine Kinase Inhibitors

- Afatinib (40 mg/day, except in Wu et al. [44], in which there were 3 doses: 40 mg/day, 30 mg/day, and 20 mg/day) [26,27,28,36,44]:

The different studies show the good tolerance of afatinib from a hematological viewpoint. Grade 3–4 cytopenia was observed in less than 4% of cases in the studies. Grade 3–4 hematological toxicities most commonly observed in older subjects treated with afatinib (affecting almost one third of patients in the studies) were mucositis (3% to 50%), diarrhea (8% to 33%), skin rash (5% to 33%), paronychia (5% to 28%), and asthenia (1% to 33%). These proportions are higher than in the general population. Likewise, the probability of developing interstitial lung disease is higher in older patients (5% to 10%). Low-grade digestive toxicities, skin toxicities, and asthenia are common. Grade 1–2 toxicities should be taken into consideration, as they contribute to the deterioration of quality of life in older people.

- Erlotinib (150 mg/day) [36,42,45,58,61,62,63,64,65,68,69,72,73,76]:

Hematological toxicities are rare in older subjects (comparable to younger subjects; 1% to 2% grade 3–4). They are essentially grade 1–2 and vary considerably between studies. The most common grade 3–4 non-hematological toxicities with erlotinib in older patients are diarrhea (3% to 17%) and skin rashes (4% to 14%). These proportions are higher than in the general population. Grade 1–2 toxicities are mainly diarrhea, anorexia, and cutaneo-mucous signs.

- Gefitinib (250 mg/day) [29,30,31,32,33,34,35,36,44,58,59,67]:

Anemia is the primary hematological toxicity retained with gefitinib; the incidence varies depending on the studies (3% to 13% for grades 3–4). The most common grade 3–4 toxicities in older patients treated with gefitinib (involving nearly 15% of patients) were an increase in AST/ALAT (7% to 50% of cases), anorexia (5% to 20% of cases), diarrhea (1% to 17% of cases), and skin rash (2% to 16% of cases). These proportions are higher than in the general population, particularly the increase in AST/ALAT and anorexia. Likewise, the incidence of interstitial lung disease was higher in older patients than in the general population. Regarding grade 1–2 toxicities, digestive, hepatic (increase in AST/ALAT, bilirubin, and PAL), and cutaneo-mucous toxicities were observed in over half of patients.

- Osimertinib (80 mg/day) [37,38,39,40,60]:

In older patients on osimertinib, there is a hematological risk of all grades and, in particular, a grade 3–4 risk of anemia (up to 43%). The most common grade 3–4 non-hematological toxicities in older subjects treated with osimertinib are anorexia (11%) and paronychia (up to 42%). These proportions are higher than in the general population. Likewise, the proportion developing interstitial lung disease is higher in older patients (5% to 10%) compared to the general population. The risk of QT prolongation should be taken into consideration, even though only one study has highlighted this risk, and an ECG should be systematically conducted before initiating osimertinib. Concerning grade 1–2 toxicities, digestive and cutaneo-mucous toxicities are common, as well as an increase in AST/ALAT and hypoalbuminemia.

- Combination of targeted therapy and chemotherapy [66,70,74,76]:

Combination of erlotinib (100 mg/day) + gemcitabine: Grade 3–4 cytopenia was observed in less than 8% of cases in the studies. Regarding non-hematological toxicities, primarily 47% of acne of all grades was reported.

Combination of erlotinib or gefitinib + chemotherapy (cisplatin, carboplatin, gemcitabine, paclitaxel, docetaxel, pemetrexed, vinorelbine, or etoposide). Regarding the toxicity data of erlotinib and gefitinib combined, almost 30% grade 1–2 skin rashes and 4% grade 3–4, and 2% grade 3–4 lung involvement, were reported. There is no toxicity more specific to older subjects for a protocol combining chemotherapy and anti-EGFR TKI.

Combination of erlotinib (150 mg/day) + bevacizumab: There is no toxicity more specific to older subjects for a protocol combining an anti-angiogenic and an anti-EGFR TKI. Particular attention must be paid to cytopenia, hemorrhagic events, hypertension, and proteinuria, but this combination is of no interest in practice.

Combination of sorafenib (800 mg/day) + gemcitabine: There is no toxicity specific to older subjects but this combination is of no interest in practice.

- Combination of erlotinib (150 mg/day) + sorafenib (800 mg/day):

There is no toxicity data specific to older subjects but this combination is of no interest in practice.

#### 3.2.2. ALK Tyrosine Kinase Inhibitors

- Alectinib (600 mg twice/day) [55]:

The tolerance profile of alectinib seems comparable to that of the general population, except for vision disorders (grades 1–2; 31% of cases), which are more common in older patients. Hematological toxicity was not reported.

- Ceritinib (450 mg/day) [55]:

The tolerance profile of ceritinib seems comparable to that of the general population, except for an increase in creatinine, more common in older patients (20% to 40% of cases). Grade 3–4 non-hematological toxicities included nausea in 20% of cases, and oedema in 8% of cases.

- Crizotinib (250 mg twice/day) [55]:

The tolerance profile of crizotinib (digestive adverse events, such as grade 3–4 nausea in 5% of cases and grade 3–4 diarrhea in 10% of cases, and grade 1–2 pulmonary adverse events in 5% of cases) seems comparable to that of the general population, except for QT prolongation (grade 1–2 in 10% of cases), which is more common in older patients.

### 3.3. Feasibility 

Table 6 and Table 7.

#### 3.3.1. EGFR Tyrosine Kinase Inhibitors

- Afatinib (40 mg/day) [26,27,28,36,77]:

Full-dose treatment in older subjects is not very feasible, and afatinib requires dose adjustments in 42% to 89% of cases. Changes in dose are primarily due to toxicities.

- Erlotinib (150 mg/day) [36,42,45,61,62,63,64,65,68,69,72,73,76]:

Full-dose treatment with erlotinib in older subjects is feasible and requires dose adjustments in 7% to 56% of cases.

- Gefitinib (250 mg/day) [29,30,31,32,33,34,35,36,44,52,58,59,67]:

Full-dose treatment with gefitinib in older subjects is feasible in most cases and requires dose adjustments in 17% to 52% of cases. In practice, there is not one single dosage. A trial will be necessary to see if intermittent treatment is required if the dose needs adjusting.

- Osimertinib (80 mg/day) [37,38,39,40,60]:

Full-dose treatment with osimertinib in older subjects is feasible in most cases and requires dose adjustments in 9% to 28% of cases.

- Erlotinib or Gefitinib + Chemotherapy [66]:

Full-dose treatment with gefitinib or erlotinib + chemotherapy in older subjects is not very feasible and requires dose adjustments in 64% of cases.

- Combination of Targeted Therapy (Sorafenib + Erlotinib) [70]:

There is little data on the feasibility of erlotinib + sorafenib and no indication in practice.

#### 3.3.2. ALK Tyrosine Kinase Inhibitors

- Alectinib (600 mg twice/day) [55]:

Little data is available regarding the feasibility of alectinib in older subjects. Nevertheless, a trial reports a 44% reduction in dose intensity.

- Ceritinib (450 mg/day) [55]:

It is difficult to any draw conclusions regarding the feasibility of ceritinib as there are so few trials including older subjects. Treatment discontinuation due to toxicity in 60% of cases was observed; thus, caution is required in older subjects.

- Crizotinib (250 mg twice/day) [55]:

It is difficult to draw any conclusions regarding the feasibility of crizotinib as there are so few trials including older subjects.

### 3.4. Quality of Life 

Only four articles [31,33,69,76] included an analysis of quality of life. One article comparing erlotinib alone or combined with chemotherapy or chemotherapy alone showed no significant difference between the three arms regarding quality of life. Another article comparing gefitinib and chemotherapy (carboplatin/paclitaxel) showed no difference between the two treatments in terms of pain, dyspnea, anxiety, and function between patients under and over 70 years. In Takahashi et al. [33], cough and breathlessness improved significantly in 20 older patients treated with gefitinib after 4 weeks of treatment. In Chen et al. [69], in a study comparing erlotinib and vinorelbine-type chemotherapy, there was no significant difference between erlotinib and chemotherapy, except for physical well-being, which was better in the erlotinib group. A priori, there was no change in quality of life of older patients on TKI compared to those undergoing chemotherapy (Table 8).

### 3.5. Geriatric Data 

In the studies included for efficacy, 20 articles included patients with an ECOG-PS ≥ 2 of which the extremes varied from 2% to 42%. The studies not including ECOG-PS ≥ 2 patients were randomized studies (subgroups of older subjects). In Kato et al. [40], according to the multivariate analysis, age and ECOG were two independent factors of treatment efficacy with osimertinib. In Brueckl et al. [64], the OS curve for 385 older patients treated with erlotinib was not significantly different according to the age groups and the ECOG-PS. Only 7 studies [36,41,42,66,71,74,76] included geriatric or frailty data in the descriptive analyses. The Charlson Comorbidity Index (CCI) was the most commonly used in the Comprehensive Geriatric Assessment (CGA), with few patients having comorbidities in most of these studies, as the percentage of CCI ≥ 3 varied from 6% to 8%. In Stinchcombe et al. [76], the CIRS-G frailty scale was used, and the median severity index was lower in the erlotinib group (compared with chemotherapy alone or the erlotinib + chemotherapy arm) but was not significantly different. Only two studies conducted a CGA [36,71]; one study [71] included patients with suspected cognitive disorders in 52% of cases (Mini Mental State Evaluation ≤ 23), loss of autonomy with activities of daily living (ADL) in 49% of cases, and a body mass index (BMI) indicating probable denutrition in 61.5% of cases. According to the logistic regression, factors associated with erlotinib treatment (second-line) in this article were PS between 0 and 1, stage IV, and an ADL index of 6. Multivariate analysis identified weight loss ≤ 5% as being associated with better OS. The other study [36] showed that 39% of patients were taking ≥6 drugs per day. In Miyamoto et al. [41], an ECOG-PS ≥ 2 was associated with a shorter PFS and OS, and a CCI ≥ 2 was associated with a shorter PFS. So, there is currently very little clinical data available to determine the geriatric profile of older patients likely to receive targeted therapy. Most of the studies included robust older patients with good PS and very few comorbidities (Table 8).

## 4. Discussions

### 4.1. For Which Older Patients with NSCLC Can We Propose the Following TKI?

#### 4.1.1. EGFR Tyrosine Kinase Inhibitors

Osimertinib, 80 mg/day, is used as a first-line treatment in older patients with the EGFR mutation (exon 19 deletion or L858R mutation). Osimertinib, 80 mg/day, is used as a second-line treatment in the case of a T790M resistance mutation in older patients pretreated with first and second generation TKI (grade C).

Full-dose treatment is feasible in older subjects but more intensive hematological monitoring than with younger patients is strongly recommended. Monitoring is recommended with osimertinib (80 mg/day): Clinical monitoring, electrocardiogram (ECG) (QT prolongation), and biological monitoring (blood count, ionogram, creatinine, AST/ALAT (Aspartate aminotransferase/Alanine aminotransferase), and AP (Alcaline phosphatase), bilirubin, albumin) (grade C) must be systematic and regular in older subjects (at least every 15 days for the first three months) (expert agreement). Given the prevalence of grade 1–2 hypoalbuminemia, nutritional status must be monitored in older subjects (at least once/month) (expert agreement).

Concerning afatinib, full-dose treatment in older subjects is not very feasible. A reduction in dose is necessary in most cases (grade A).

Clinical and biological monitoring (blood count, ionogram, creatinine, AST/ALAT) (grade B) are necessary with afatinib (40 mg/day) and must be systematic and regular for older subjects (at least every 15 days for the first three months) (expert agreement).

Full-dose treatment with erlotinib is feasible (grade B). Clinical and biological monitoring (blood count, ionogram, creatinine, AST/ALAT) are necessary with erlotinib (150 mg/day) (grade C) and must be systematic and regular for older subjects (at least every 15 days for the first three months) (expert agreement).

Full-dose treatment with gefitinib is feasible (grade C). Clinical and biological monitoring (blood count, ionogram, creatinine, AST/ALAT, AP, bilirubin) are required with gefitinib (250 mg/day) (grade C) and must be systematic and regular for older subjects (at least every 15 days for the first three months) (expert agreement).

#### 4.1.2. ALK Tyrosine Kinase Inhibitors

In older patients with an ALK rearrangement, there is very scarce data to enable specific recommendations to be established. The recommendations are the same as for younger patients: Alectinib 600 mg twice/day is given as a first-line treatment, but beware of the risk of dose reduction with alectinib, and also brigatinib 90 mg per day for the first 7 days and then 180 mg per day (grade C). Phase IV data are necessary to draw conclusions regarding the feasibility of this treatment in an older population. Alectinib (600 mg/day) requires clinical (in particular, ophthalmic) and biological monitoring (blood count, ionogram, creatinine, AST/ALAT) (grade C) and must be systematic and regular for older subjects (at least every 15 days for the first three months) (expert agreement).

Clinical, ECG, and biological monitoring (blood count, ionogram, creatinine, AST/ALAT) are required with ceritinib (450 mg/day) (grade C) and must be systematic and regular for older subjects (at least every 15 days for the first three months) (expert agreement).

Clinical, ECG, and biological monitoring (blood count, ionogram, creatinine, AST/ALAT) are required with crizotinib (250 mg twice/day) (grade C) and must be systematic and regular for older subjects (at least every 15 days for the first three months) (expert agreement).

#### 4.1.3. The ROS Tyrosine Kinase Inhibitor Found in this Systematic Review in Older Subjects: Crizotinib

Regarding ROS1 rearrangements, there is very little data to enable specific recommendations to be issued for older subjects. The recommendations are the same as for younger subjects: Crizotinib 250 mg twice a day is given as a first-line treatment (grade B). Phase IV trials are necessary. Clinical, ECG, and biological monitoring (blood count, ionogram, creatinine, AST/ALAT) are required with crizotinib (250 mg twice/day) (grade B) and must be systematic and regular for older subjects (at least every 15 days for the first three months) (expert agreement).

### 4.2. For Which Older Patients with NSCLC Should We Consider Monotherapy (TKI Alone)?

For the time being, given the data in the literature regarding older subjects treated with TKI, there is no indication to combine TKI with chemotherapy or another systemic treatment. TKI is to be prescribed as monotherapy in this population (expert agreement). Moreover, there is no marketing authorization for combinations of chemotherapy and TKI for NSCLC.

### 4.3. For Which Older Patients with NSCLC Can We Consider a Combination of Several TKI?

Even though TKI combination is not yet approved by the Food and Drugs Administration or the European Medicines Agency (EMA), some studies have already explored the possibility of combination treatment with TKI [77,78]. For the time being, given the lack of data in the literature on older subjects treated with TKI, there is no indication to combine several TKI in this population for the moment (expert agreement).

### 4.4. General Recommendations for Prescribing TKI for the Treatment of NSCLC in Older Patients

Regarding the conditions for prescribing TKI in older subjects (≥70 years), a CGA can help identify de-nutrition, comorbidities, and an alteration of functional status, which have an impact on overall survival and progression-free survival (grade B), and propose corrective action. So, a CGA must be recommended for older patients with a G8 ≤ 14/17 according to the recommendations [79], but it is also important to assess nutritional status through changes in weight or BMI or MNA (Mini Nutritional Assessment) [80,81], functional status through ADL (Activities of Daily Living) [81,82], and comorbidities using the Charlson index [83] or CIRS-G (Cumulative Illness Rating Scale-Geriatric) [84] before prescribing TKI in older patients with NSCLC (expert agreement).

Targeted therapy does not seem to have a greater impact on quality of life than other systemic treatments but must be monitored regularly (grade B). In view of the literature studied, the work group does not recommend any particular tool to measure quality of life (expert agreement).

## 5. Conclusions

This is the first review in the current literature grouping two learned societies (SPLF/GOLF and SoFOG) and bringing together experts in pulmonary-oncology and geriatric oncology on the subject of prescribing targeted therapy in older patients with NSCLC. These recommendations are based on 52 articles included from May 2010 to May 2020 and after revision between April 2020 and May 2021. The selection process is documented in a PRISMA flow diagram. Regarding EGFR tyrosine kinase inhibitors, first generation molecules, such as erlotinib and gefitinib, can be used at full dose but afatinib, a second generation TKI, is not feasible at full dose in older subjects. Osimertinib (3rd generation TKI) at a dose of 80 mg/day seems to be the best treatment option for older patients with the EGFR mutation (exon 19 deletion or L858R mutation) and as a second-line treatment in the case of a T790M resistance mutation. Hematological monitoring must be reinforced with this treatment. For ALK and ROS1 tyrosine kinase inhibitors, the lack of data prevents specific recommendations being established for older subjects. Given the data in the literature, targeted therapies must not be combined together or with chemotherapy, for the time being. Finally, a geriatric assessment is recommended after conducting a G8-type test to identify frailty (if ≤14/17), as well as to identify comorbidities that could interfere with the good progress of the treatment, loss of functional status, and alteration of nutritional status. Polypharmacy (≥5 drugs) also needs to be investigated to request the help of pharmacists in identifying any interactions between regular medications and the targeted therapy initiated.

## Figures and Tables

**Table 1:** Targeted therapy efficacy results in older patients in retrospective and prospective cohorts (≥65 years).

Publication/Country	Targeted Therapy	Patient Number	ECOG-PS ≥ 2 (%)	Median Age, Years	Objective Tumor Response (95% CI)	Median PFS, Months (95% CI)	Median OS, Months (95% CI)
Tanaka 2018 [26]/Japan	Afatinib 40 mg/day 30 mg/day 20 mg/day	15	13.3	79	73.3 (NR)	22 (13.1-NR)	
Imai 2018 [27]/Japan	Afatinib 30 mg/day	40	2.5	77	72.5 (58.6–86.3)	12.9 (8.8–19.3)	NR At 1 year: 87.4%, 2 years: 60.6%
Minegishi 2021 [28]/Japan	Afatinib 40 mg/day	37	0	77.5	75.7 (58.8–88.2)	14.2 (9.5–19.0)	35.2 (35.2-NR) At 1 year: 83.8%, 2 years: 78.3%
Tateishi 2013 [29]/Japan	Gefitinib 250 mg/day	55	16.4	81.1	72.7 (59.5–82.9)	13.8 (9.9–18.8)	29.1 (22.4-NR) At 2 years: 59.5%
Fujita 2012 [30]/Japan	Gefitinib 250 mg/day	54	0	81	45.5 (24.4–67.8)		27.9 At 1 year: 90%
Morikawa 2015 [31]/Japan	Gefitinib 250 mg/day versuscaboplatine/ paclitaxel	71	8	75	73.2 (61.3–83.0)	14.3	30.8
Maemondo 2012 [32]/Japan	Gefitinib 250 mg/day	31	6	80.3	74.2 (57.9–90.5)	12.1	33.8 At 1 year: 83.9%, at 2 years: 58.1
Takahashi 2014 [33]/Japan	Gefitinib 250 mg/day	20	10	79.5	70 (45.7–88.1)	10.0	26.4
Kuwako 2015 [34]/Japan	Gefitinib 250 mg/day	62	29	80	61.3	13.2	19
Asami 2011 [35]/Japan	Gefitinib 250 mg/day	17	17	81	59 (33–81)	12.9 (2.2–23.6)	OS (NR) At 1 year: 88%
Corre 2018 [36]/France	Gefitinib or erlotinib or afatinib	114	28.4	83.9	63.3	11.9 (8.6–14.7)	20.9 (14.3–27.1)
Furuta 2018 [37]/Japan	Osimertinib	18	0	80	61	17.7 (8.4-NR)	38.6 (14.3–52.8)
Nakao 2020 [38]/Japan	Osimertinib 80 mg/day	36	0	80	58.3 (42.2–72.9)	11.9 (7.9–17.5)	22.0 (16.0-NR) at 1 year: 77.8%, at 2 years: 49.5%
Auliac 2019 [39]/France	Osimertinib 80 mg/day	43	42.4	84.6		17.5 (12.2–19.0)	22.8 (15.7-NR)
Kato 2019 [40]/Japan	Osimertinib	31	10	32.3		5.6 (3.6–14.8)	19.4 (9.1-NR)
		8	3	54		3.5 (1.6–14.8)	5.3
		23	7	75		6.4 (5-NR) HR 2.41; *p* = 0.041	19.4 HR 2.58; *p* = 0.067
Miyamoto 2020 [41]/Japan	Erlotinib 50 mg/day	80	32	80	60.0 (50.2–69.2)	9.3 (7.2–11.4)	26.2 (21.9–30.4)
Inoue 2015 [42]/Japan	Erlotinib 150 mg/day	32	3.1	80	56.3 (39.4–72.0)	15.5 (11.2-NR)	Median OS (NR) At 1 year: 83.9% (65.5–93.0)

HR: hazard ratio; ECOG-PS: eastern cooperative oncology group-performance status; NR: not reached; PFS: progression-free survival; OS: overall survival. For each treatment, when several studies express results as medians with intervals, the minimum and maximum medians observed are reported in the table. When there is only one study for a given treatment, the median with its interval is reported in the table.

**Table 2:** Targeted therapy efficacy results in older patients in randomized or non-randomized trials (subgroup post-hoc analysis).

Publication/ Country	Targeted Therapy	Patients Number	Age Group	ECOG-PS ≥ 2 (%)	PFS (Months) (95% CI)	OS (Months) (95% CI)
Park 2016 [43]/ International	Afatinib 40 mg/day or gefitinib 250 mg/day	319		0		
		177	<65		afatinib 11.0 (9.2–17.0) gefitinib 9.2 (7.3–11.0) HR 0.68 (0.48–0.97)	
		142	≥65		afatinib 11.0 (9.2–12.9) gefitinib 11.4 (10.8–12.9) HR 0.85 (0.59–1.22); *p* = 0.309	
Wu 2018 [44]/ International	Afatinib 40 mg/day or gefitinib 250 mg/day	319		0		
		177	<75		Afatinib 11.0 Gefitinib 10.9 HR 0.76 (0.58–1.00)	Afatinib 28.9 Gefitinib 25.2 HR 0.85 (0.64–1.12)
		142	≥75		Afatinib 14.7 Gefitinib 10.8 HR 0.69 (0.33–1.44)	Afatinib 27.9 Gefitinib 19.7 HR 1.05 (0.5–2.21)
Paz-Ares 2017 [53]/International	Afatinib 40 mg/day or gefitinib 250 mg/day	319		0		
		177	<65			0.66 (0.46–0.94) HR 1.22 (0.82–1.81); *p* = 0.0228
		142	≥65			
Sequist 2013 [45]/ International	Afatinib 40 mg/day or cisplatine/pemetrexed	345				
		211	<65		HR 0.53 (0.36–0.76)	
		134	≥65		HR 0.64 (0.39–1.03); *p* = 0.58	
Wu 2017 [46]/International	Dacomitinib 45 mg/day or gefitinib 250 mg/day	452		0		
		dacomitinib/ gefitinib	<65		HR 0.51 (0.39–0.69)	
		dacomitinib/ gefitinib	≥65		HR 0.69 (0.48–0.99)	
Zhou 2011 [47]/China	Erlotinib 150 mg/dayor gemcitabine/cisplatin	165		9		
		64	<65		HR 0.19 (0.11–0.31)	
		19	≥65		HR 0.17 (0.07–0.43)	
Rosell 2012 [48]/International	Erlotinib 150 mg/day or cisplatin/docetaxel or gemcitabin	173		14		
		85	<65		HR 0.44 (0.25–0.75)	
		88	≥65		HR 0.28 (0.16–0.51) *p* = 0.4962	
Soria 2018 [49]/International	Osimertinib 80 mg/day or gefitinib 250 mg/day or erlotinib 150 mg/day	556		0		
		298	<65		HR 0.44 (0.33–0.58)	
		258	≥65		HR 0.49 (0.35–0.67)	
Ramalingam 2020 [50]/International	Osimertinib 80 mg/day or gefitinib 250 mg/day or erlotinib 150 mg/day	556		0		
		298	<65			HR 0.72 (0.54–0.97)
		258	≥65			HR 0.87 (0.63–1.22)
Mok 2017 [51]/ International	Osimertinib 80 mg/day	279				
		242	<65		HR 0.38 (0.28–0.54)	
		177	≥65		HR 0.34 (0.23–0.50)	
Douillard 2014 [52]/International	Gefitinib 250 mg/day	106		6.6		
		55	≤65		65.5 (52.3–76.6)	
		51	> 65		74.5 (61.1–84.5)	

HR: hazard ratio; ECOG-PS: eastern cooperative oncology group-performance status; NR: not reached; PFS: progression-free survival; OS: overall survival. For each treatment, when several studies express results as medians with intervals, the minimum and maximum medians observed are reported in the table. When there is only one study for a given treatment, the median with its interval is reported in the table.

**Table 3:** Targeted therapy efficacy results in older patients in randomized or non-randomized trials (subgroup post-hoc analysis).

Publication/ Country	Targeted Therapy	Patient Number	Age Group	ECOG-PS ≥ 2 (%)	Objective Response Rate (95% CI)	PFS (Months) (95% CI)	OS (Months) (95% CI)
Hida 2017 [54]/ Japan	Alectinib 300 mg twice a day or crizotinib 250 mg twice a day	207		2			
		185	<75			HR 0.34 (0.21–0.56)	
		22	≥75			HR 0.28 (0.06–1.19)	
Peters 2017 [16]/ International	Alectinib 600 mg twice a day or crizotinib 250 mg twice a day	303		7			
		233	<65			HR 0.48 (0.34–0.70)	
		70	≥65			HR 0.45 (0.24–0.87)	
Camidge 2012 [56]/International	Crizotinib 250 mg twice a day	149		12			
		123	<65		60.2 (50.9–68.9)		
		20	≥65		65.0 (40.8–84.6)		
Soria 2017 [17]/ International	Ceritinib 750 mg/day or cisplatin/pemetrexed	376		0			
		295	<65			17.1 (12.5–27.7) HR 0.58 (0.42–0.80)	
		81	≥65			14.0 (8.3-NR) HR 0.45 (0.24–0.86)	
Bedas 2019 [55]/ Israel	Crizotinib or alectinib or ceritinib	53		11		crizotinib 5.6 (2.5–14.7)	25.1 (10.8–53.6)
		34	<65			ceritinib 23 (0.8–27.7)	
		19	≥65			alectinib 5.6 (0.5-NR)	

HR: hazard ratio; ECOG-PS: eastern cooperative oncology group-performance status; NR: not reached; PFS: progression-free survival; OS: overall survival. For each treatment, when several studies express results as medians with intervals, the minimum and maximum medians observed are reported in the table. When there is only one study for a given treatment, the median with its interval is reported in the table.

**Table 4:** Prevalence of hematological and biological toxicities according to targeted therapies in trials, cohorts, or trial subgroup post-hoc analysis.

Molecules	Afatinib	Gefitinib	Osimertinib	Crizotinib	Ceritinib	Alectinib	Erlotinib	Combination of TKI	Combination of TKI and Chemotherapy
Publications	Tanaka 2018 [26], Imai 2018 [27], Wu 2018 [44], Corre 2018 [36], Minegishi 2021 [28]	Wu 2018 [44], Inomata 2016 [58], Tateishi 2013 [29], Fujita 2012 [30], Morikawa 2015 [31], Maemondo 2012 [32], Takahashi 2014 [33], Kuwako 2015 [34], Asami 2011 [35], Corre 2018 [36], Wu 2015 [59], Kobayashi 2011 [67]	Furuta 2018 [37], Nakao 2020 [38], Nakao 2019 [60], Auliac 2019 [39], Kato 2019 [40]	Bedas 2019 [55]	Bedas 2019 [55]	Bedas 2019 [55]	Minemura 2015 [61], Stinchcombe 2011 [76], Rossi 2010 [62], Inomata 2016 [58], Yoshioka 2014 [63], Merimsky 2012 [72], Kurishima 2013 [73], Brueckl 2018 [64], Corre 2018 [36], Heigener 2014 [68], Chen 2012 [69], Inoue 2015 [42], Yamada 2016 [65], Quoix 2014 [71]	Gridelli 2011 [70]	Gridelli 2011 [70], Stinchcombe 2011 [76], Aoshima 2020 [66], Tam 2013 [74]
Anemia (%)									
Grade 1–2	4–60	6–50	28–75				6–80	3	3–12
Grade 3–4	2	3–13	6–43						8
Leucopenia (%)									
Grade 1–2	2–3	4–10	17–36				3–20		16
Grade 3–4	1–2		3–17						
Neutropenia (%)									
Grade 1–2	3–17	1–3	39				3–10		19
Grade 3–4	1–2		3–6				1–2		2–3
Thrombocytopenia (%)									
Grade 1–2	21	1–10	56–58				17.5	3	3
Grade 3–4	2		3				2		4–10
AST/ALT elevation (%)									
Grade 1–2	5–33	10–60	22–36	16	20	22	8–37.5	6	6–20
Grade 3–4	5	7–50	6				1–6		2
Bilirubin elevation (%)									
Grade 1–2	3	10–13	8				40	6	
Grade 3–4		3							
AP elevation (%)									
Grade 1–2			25–34						
Grade 3–4		27	6						
Creatinine elevation (%)									
Grade 1–2	17	13–16	25–31	16	40		6–40		9–12
Grade 3–4									
Grade 5							1		2
Hypoalbuminemia (%)									
Grade 1–2		41	69–75						
Grade 3–4			3						
Amylase-lipase elevation (%)									
Grade 1–2									
Grade 3–4	3							3	
Hyperkalemia									
Grade 1–2 (%)	23								

AST = aspartate aminotransferase; ALT = alanine aminotransferase; AP = alkaline phosphatase; TKI: Tyrosine kinase inhibitors. For each treatment, when multiple studies report results by type of toxicity and grade, the minimum percentage observed and the maximum percentage observed in these studies are reported in the table. When there is only one study for a given treatment, the percentage observed in the study is reported according to the type of toxicity and its grade.

**Table 5:** Prevalence of non-hematological and non-biological toxicities according to targeted therapies in trials, cohorts, or trial subgroup post-hoc analysis.

Molecules	Afatinib	Gefitinib	Osimertinib	Crizotinib	Ceritinib	Alectinib	Erlotinib	Combination of TKI	Combination of TKI and Chemotherapy
Publications	Tanaka 2018 [26], Imai 2018 [27], Wu 2018 [44], Corre 2018 [36], Minegishi 2021 [28]	Wu 2018 [44], Inomata 2016 [58], Tateishi 2013 [29], Fujita 2012 [30], Morikawa 2015 [31], Maemondo 2012 [32], Takahashi 2014 [33], Kuwako 2015 [34], Asami 2011 [35], Corre 2018 [36], Wu 2015 [59], Kobayashi 2011 [67]	Furuta 2018 [37], Nakao 2020 [38], Nakao 2019 [60], Auliac 2019 [39], Kato 2019 [40]	Bedas 2019 [55]	Bedas 2019 [55]	Bedas 2019 [55]	Minemura 2015 [61], Stinchcombe 2011 [76], Rossi 2010 [62], Inomata 2016 [58], Yoshioka 2014 [63], Merimsky 2012 [72], Kurishima 2013 [73], Brueckl 2018 [64], Corre 2018 [36], Heigener 2014 [68], Chen 2012 [69], Inoue 2015 [42], Yamada 2016 [65], Quoix 2014 [71]	Gridelli 2011 [70]	Gridelli 2011 [70], Stinchcombe 2011 [76], Aoshima 2020 [66], Tam 2013 [74]
Nausea (%)									
Grade 1–2	8–50	2–19		42	60	22	2	6	16–24
Grade 3–4	3–17	2–3		5	20		1		16
Vomiting (%)									
Grade 1–2	5–50	1–23					1–7.5	6	
Grade 3–4	2–3	5					1		
Anorexia (%)									
Grade 1–2	17–33	13–50	28–31				12.5–50	14	3
Grade 3–4	3–17	5–20	11				6		
Dysgeusia (%)									
Grade 1–2							6		15
Grade 3–4									
Asthenia/fatigue (%)									
All grades							17		
Grade 1–2	13–67	6–40	28–31	32	40	44	2–42.5	28	19–12
Grade 3–4	1–33	3	8–9	5			2–5	14	13–10
Diarrhea (%)									
All grades							30		
Grade 1–2	67–100	6–52	22–39	32	60		12.5–80	38	9–32
Grade 3–4	8–33	1–17	2.8	10			3–17	17	3–6
Grade 5		17							
Skin rash (%)									
All grades							69		
Grade 1–2	33–74	31–90	22–36				3–95	35	26–30-60
Grade 3–4	5–33	2–16					4–14	13	4–6–16
Acne (%)									
All grades									47
Grade 1–2							45		
Grade 3–4							31		
Paronychia (%)									
Grade 1–2	26–50	19–30	33				6–37.5	3	3–36
Grade 3–4	5–28	4–5	17–42						
Mucositis-stomatitis (%)									
Grade 1–2	31–60	1–24	17–22				6–12.5–28		16–19
Grade 3–4	3–50	3–8							
Dry skin (%)									
Grade 1–2	9–38	8–65					6–59		
Grade 3–4							3–5		
Pruritus (%)									
Grade 1–2	14–26	6–24	22				62.5		3
Grade 3–4	1–2						2.5		
Urticaria (%)									
Grade 1–2	15								
Grade 3–4									
Edema (%)									
Grade 1–2	10.5			37		33			
Grade 3–4				26	8		1		
Infection (%)									
Grade 1–2	3–17						1		
Grade 3–4	3–17						1		
Interstitial lung disease (%)									
Grade 1–2	8	1–6	3	5			1		
Grade 3–4	5–10	2–4	6–9				1–6		2
Grade 5									2
Constipation (%)									
Grade 1–2	3–4	6.5		10					
Grade 3–4									
Dehydration (%)									
Grade 1–2									
Grade 3–4			3				1–6		6
Alopecia (%)									
Grade 1–2		6–10					19	3	6
Grade 3–4									
Pigmentation (%)									
Grade 1–2		21.6							
Faintness (%)									
Grade 1–2		12					6		
Grade 3–4							1		
Ventricular dysfunction (%)									
Grade 3–4			3						
Prolonged QT interval (%)									
Grade 1–2				10					
Grade 3–4			3						
Hand-foot syndrome (%)									
Grade 1–2							27.5	21	20
Grade 3–4			3					10	
Delirium (%)									
Grade 3–4			3						
Dyspnea (%)									
All grades							17.5		
Grade 3–4			3						6
Sinusitis (%)									
Grade 3–4			3						
Fever (%)									
Grade 1–2			11						3–8
Vision disturbances (%)									
Grade 1–2				31		31	9.7		
Conjunctivitis (%)									
Grade 3–4							1		
Neuropathy (%)									
Grade 3–4							1		
Erythema multiform (%)									
Grade 1–2							25		
Grade 3–4							7.5		
Dizziness (%)									
Grade 1–2							3		
Grade 3–4							1		
Proteinuria (%)									
Grade 1–2									20
Grade 3–4									8
Arterial hypertension (%)									
Grade 1–2								7	3–16
Intracranial hemorrhage (%)									
Grade 1–2									4
Epistaxis (%)									
Grade 1–2									4
Gastrointestinal bleeding (%)									8–12
Gastric perforation (%)									
Grade 3–4									4
Pneumothorax (%)									
Grade 3–4									4
Pneumonia (%)									
Grade 1–2									8
Cardiac toxicity (%)									
Grade 3–4								3	3
Colonic perforation (%)									
Grade 3–4									3
Dysphonia (%)									
Grade 1–2									3
Endobronchial cavitation (%)								3	
Hemorrhages (%)									
Grade 1–2								3	
Grade 3–4								3	

TKI: Tyrosine kinase inhibitors. For each treatment, when multiple studies report results by type of toxicity and grade, the minimum percentage observed and the maximum percentage observed in these studies are reported in the table. When there is only one study for a given treatment, the percentage observed in the study is reported according to the type of toxicity and its grade.

**Table 6:** Targeted therapy feasibility results in older patients in non-randomized trials.

Molecules	Afatinib	Gefitinib	Osimertinib	Crizotinib	Ceritinib	Alectinib	Erlotinib	Combination of TKI and Chemotherapy
Publications	Tanaka 2018 [26], Imai 2018 [27], Corre 2018 [36], Minegishi 2021 [28]	Douillard 2014 [52], Inomata 2016 [58], Tateishi 2013 [29], Fujita 2012 [30], Morikawa 2015 [31], Maemondo 2012 [32], Takahashi 2014 [33], Kuwako 2015 [34], Asami 2011 [35], Corre 2018 [36], Wu 2015 [59], Kobayashi 2011 [67]	Furuta 2018 [37], Nakao 2020 [38], Nakao 2019 [60], Auliac 2019 [39], Kato 2019 [40]	Bedas 2019 [55]	Bedas 2019 [55]	Bedas 2019 [55]	Minemura 2015 [61], Stinchcombe 2011 [76], Rossi 2010 [62], Yoshioka 2014 [63], Merimsky 2012 [72], Kurishima 2013 [73], Brueckl 2018 [64], Corre 2018 [36], Heigener 2014 [68], Chen 2012 [69], Inoue 2015 [42], Yamada 2016 [65]	Aoshima 2020 [66], Tam 2013 [74]
Median duration of treatment (months)	4.0 (1–69)	1.6–8.0	15.0 ± 9	4.2	5.8	5.0	1–39 1–9 1.7–6.2	10.4
Dose reduction (%)	47.5–89	20–45	19–39	21	60	44	7–56	64
Treatment discontinuation due to toxicity (%)	5–21	3–52	9–28	21	60		4–45	
Dose reduction due to toxicity (%)		17	9–28				7–56	64

TKI: Tyrosine kinase inhibitors. For each treatment, when several studies express results as medians with intervals, the minimum and maximum medians observed are reported in the table. When there is only one study for a given treatment, the median with its interval is reported in the table. For each treatment, when multiple studies report results by dose reduction and treatment discontinuation, the minimum percentage observed and the maximum percentage observed in these studies are reported in the table. When there is only one study for a given treatment, the percentage observed in the study is reported according to dose reduction or treatment discontinuation.

**Table 7:** Targeted therapy feasibility results in older patients in randomized trials.

Molecules	Afatinib	Gefitinib	Osimertinib	Crizotinib	Ceritinib	Alectinib	Erlotinib	Combination of TKI
Publications	Wu 2018 [44]	Wu 2018 [44]					Quoix 2013 [71]	Gridelli 2011 [70]
Median duration of treatment (months)	12	12					2.0–2.2	
Dose reduction (%)	42							
Treatment discontinuation due to toxicity (%)	9	14					12	21
Dose reduction due to toxicity (%)	42	29						

TKI: Tyrosine kinase inhibitors. For each treatment, when several studies express results as medians with intervals, the minimum and maximum medians observed are reported in the table. When there is only one study for a given treatment, the median with its interval is reported in the table. For each treatment, when multiple studies report results by dose reduction and treatment discontinuation, the minimum percentage observed and the maximum percentage observed in these studies are reported in the table. When there is only one study for a given treatment, the percentage observed in the study is reported according to dose reduction or treatment discontinuation.

**Table 8:** Geriatric assessment and quality of life results in older patients in randomized and non-randomized trials.

Publication	Treatment	Median Age (Years)	Comorbidities-Charlson Scale (CCI) or Frailty Scales (%)	Quality of Life	CGA
Aoshima 2020 [66]	Erlotinib 150 mg/day + bevacizumab	80	CCI = 1: 36%, CCI = 2: 4%, CCI ≥ 3: 8%		
Stinchcombe 2011 [76]	Erlotinib 100 mg/day + chemotherapy or erlotinib alone (150 mg/day) or chemotherapy alone	76	CIRS-G frailty scale	No differences in quality of life	
Morikawa 2015 [31]	Gefitinib 250 mg/day or carboplatin/paclitaxel	75		No differences in the quality of life domains of pain and dyspnea, anxiety, and daily functioning between <70 and >70 years groups	
Takahashi 2014 [33]	Gefitinib 250 mg/day	79.5		Shortness of breath and cough improved significantly after 4 weeks of treatment	
Miyamoto 2020 [41]	Erlotinib 50 mg/day	80	CCI ≥ 6 was the cut off for frailty		
Corre 2018 [36]	Gefitinib or erlotinib or afatinib	83.9			CGA was performed for 35% of patients
Inoue 2015 [42]	Erlotinib 150 mg/day	80	CCI 1–2: 44% CCI ≥ 3: 6%		
Chen 2012 [69]	Erlotinib 150 mg/day or vinorelbine	77		Patients in the erlotinib arm had significantly better physical well-being than patients in the vinorelbine arm	
Quoix 2013 [71]	Erlotinib 150 mg/day (second line)		CCI ≤ 2: 68%		MMSE < 24: 52% ADL < 6: 49% BMI < 21: 61.5%
Tam 2013 [74]	Erlotinib or gefitinib (first or second line)	73	CCI = 1: 18%, CCI = 2: 9%		

ADL: activities of daily living; CIRS-G: cumulative illness rating scale; CCI: Charlson scale; CGA: comprehensive geriatric assessment; BMI: body mass index; MMSE: mini mental state examination.

## Supplementary Materials

The following supporting information can be downloaded at: https://www.mdpi.com/article/10.3390/cancers14030769/s1, Figure S1: MeSH terms; Table S1. Table of the recommendations staging (grade A, B or C) translated from [25].
