# Supplementary material for: Targeted Therapy for Older Patients with Non-Small Cell Lung Cancer: Systematic Review and Guidelines from the French Society of Geriatric Oncology (SoFOG) and the French-Language Society of Pulmonology (SPLF)/French-Language Oncology Group (GOLF)
Source: Cancers (Basel). 2022 Feb 2;14(3):769. doi: 10.3390/cancers14030769 (PMC8834005; doi:10.3390/cancers14030769)
Supplement: Supplementary file 1 [file cancers-14-00769-s001.zip › cancers-1543208-supplementary.pdf]

Figure S1. MeSH Terms

Indications: (TITLE)

"non small cell lung cancer" OR "lung neoplasm*" OR "NSCLC" OR "lung cancer*" OR "lung tumor*" OR “lung adenocarcinoma” OR “adenocarcinoma of the lung”

Population: (TITLE)

aged OR senior OR elderly OR frailty OR older OR octogenarian* OR 65 OR 70 OR 75 OR 80 OR 85 OR 90 OR "Nonagenarian*"

Treatment: (TITLE/ABSTRACT)

"targeted agents" OR "molecular targeted therapy" OR erlotinib OR gefitinib OR afatinib OR dacomitinib OR osimertinib OR bevacizumab OR alectinib OR ceritinib OR crizotinib OR dabrafenib OR trametinib OR vemurafenib OR capmatinib OR merestinib OR savolitinib OR brigatinib OR lorlatinib OR nintedanib OR ramucirumab OR trastuzumab OR tepotinib OR entrectinib OR repotrectinib OR pralsetinib OR selpercatinib OR larotrectinib OR "Tyrosine Kinase Inhibitor*" or "TKI*" OR "monoclonal antibod*"

Criteria: (TITLE / ABSTRACT)

"adverse events" OR "side effects" OR toxicity OR safety OR "overall survival" OR "progression-free survival" OR "quality of life" OR"response rate" OR efficacy OR QOL e of Site. Available online: URL (accessed on Day Month Year).

Table S1. Table of the recommendations staging (grade A, B or C) translated from [25]

Recommendation Grades	Proof level provided by literature
A<br>Established proof	Level 1<br>- Comparative randomized studies with high power<br>- Meta-analysis of comparative randomized studies<br>- Decision analysis based on well lead studies
B<br>Scientific presumption	Level 2<br>- Comparative randomized studies with low power<br>- Comparative non randomized, well lead studies<br>- Cohort studies
C<br>Low scientific proof	Level 3<br>- Case control studies<br>Level 4<br>- Comparative studies with important biases;<br>- Retrospective studies<br>- Cases series<br>- Descriptive epidemiologic studies (transversal, longitudinal)
